# Feasibility and Accuracy of Different Methods for Collecting Data on Falls Among Older People With Dementia

**DOI:** 10.1097/WAD.0000000000000364

**Published:** 2019-11-26

**Authors:** Natalia Adamczewska, Michael Vassallo, Peter W. Thomas, Sarah Thomas, Yolanda Barrado-Martín, Samuel R. Nyman

**Affiliations:** * Department of Psychology and Ageing & Dementia Research Centre; † Centre of Postgraduate Medical Research and Education; ‡ Bournemouth University Clinical Research Unit, Bournemouth University, Bournemouth, Poole Dorset, UK

**Keywords:** dementia, fall, self-report, trial

## Abstract

This study compared different methods for collecting data on falls among people with dementia to identify which is most feasible and accurate. Eighty-three dyads, comprised of a community-dwelling person with dementia and their informal carer, participated in the TAi ChI for people with demenTia (TACIT) trial. Falls were collected prospectively over 6 months using monthly calendars, weekly and monthly telephone interviews, and 3-monthly telephone interviews with the carer. Unique falls identified across the reporting methods were combined, and this total was compared against each reporting method in isolation and combinations. A higher frequency of falls indicated greater accuracy. Falls data collection was most feasible with weekly telephone interviews (84%), and most accurate with the combination of weekly telephone interviews with monthly calendars (96%). For the greatest completeness and accuracy of falls data with community-dwelling people with dementia, researchers should use both weekly telephone interviews and monthly calendars.

## Abstract 2

Supplemental Digital Content is available in the text.

Falls are highly prevalent among older people and can have devastating consequences such as disability and increased risk of mortality.^1^ For people with cognitive impairment, they have an increased risk of falls and injurious falls compared with their peers with preserved cognition.^2^ Effective interventions to prevent falls are required, yet to evaluate such interventions, there is a need to obtain complete and accurate falls data.

It is recommended that older people report the incidence of falls daily in a diary or calendar to be returned monthly and to collect further/missing falls data by interview.^3^ However, little is known about the feasibility and accuracy of fall reporting methods with older people with cognitive impairment. Only 1 study has explored the accuracy of fall reporting methods with older people with dementia (PWD).^4^ This study recruited patients from geriatric rehabilitation wards and out-patient nursing services, and so their data may not generalize to general community-dwelling PWD given their higher fall risk and morbidity profile.^5^ To inform future evaluations of interventions to prevent falls among PWD, the aim of the present study was to compare the feasibility and accuracy of different methods for collecting falls from PWD and their informal carers. To our knowledge, this is the first study to assess these parameters among community-dwelling PWD.

## METHODS

### Design

This study used data from a randomized controlled trial investigating the effectiveness of Tai Chi to improve postural balance among community-dwelling PWD.^6^ The study was approved by the West of Scotland Research Ethics Committee 4 (reference: 16/WS/0139) and the Health Research Authority (IRAS project ID: 209193).

### Setting

The study was conducted in 3 locations across the South of England. Participants were recruited via various sources such as National Health Service memory clinics, local charities, and self-referral. The intervention group received a Tai Chi exercise intervention for 20 weeks in addition to usual care, while the control group received usual care only. Irrespective of the random group allocation, all participants recorded their falls in the same way.

### Participants

We recruited dyads into the study, comprising a person with dementia and an informal carer. PWD who were eligible were: aged 18 or above, community-dwelling, had a diagnosis of a dementia (indicated on their medical record held by the NHS or general practitioner), physically able to do standing Tai Chi, and willing to attend weekly Tai Chi classes. Several exclusion criteria were applied: living in a care home; in receipt of palliative care; severe dementia (score of 0 to 9 on the Mini Addenbrooke’s Cognitive Examination)^7^; diagnosis of Lewy body dementia or dementia with Parkinson’s disease; severe sensory impairment; currently or within the past 6 months practising Tai Chi (or similar exercise—Qi Gong, yoga or Pilates) weekly or more; under the care of, or referred to, a falls clinic for assessment; currently attending a balance exercise programme (eg, Otago classes); or lacking mental capacity to provide informed consent. Details of the carers are provided in Table S1 (Supplemental Digital Content 1, http://links.lww.com/WAD/A256).

### Procedure

Falls were defined as, “an unexpected event in which the participants come to rest on the ground, floor or lower level.”^3^ Falls for PWD were recorded prospectively on a daily basis using monthly calendars and returned by post at the end of each month. Calendars were deemed invalid if they were returned blank or ineligible. A researcher conducted telephone interviews that were planned to be on a weekly (7-day recall) and monthly (monthly recall) basis with the PWD and every 3 months with the carer. However, in practice, some weekly and monthly telephone interviews with PWD were not possible because either the person with dementia or their informal carer insisted that their carer provided the falls data.

### Statistical Analysis

#### Feasibility of Falls Data Collection

The feasibility of different self-report methods was assessed by summarizing and comparing the expected volume of data for each method with the data actually collected. The number of weekly interviews expected was calculated from the total number of days each dyad participated in the study divided by 7. Numbers of monthly interviews and calendars expected were manually calculated from calendar dates, given that monthly falls reporting was at the end of each calendar month. Data on falls were categorized as missing if no specific date could be confirmed for the reported fall, and therefore could not be verified as a new/duplicate fall in relation to falls already reported by other methods (see the accuracy of fall reporting section below). This type of missing data was only relevant for the telephone interview data. The percentage of falls with missing date data was compared across the different methods and between PWD and carers.

#### Accuracy of Fall Reporting

In line with a previous approach,^4^ given that falls are under-reported, we assumed that methods that provide lower frequencies of falls are less accurate. Thus, the criterion variable for the total number of falls was calculated as the highest number of unique fall events that occurred when each separate method of data collection was converged, removing duplicates. This criterion variable of the total number of falls was then used to descriptively compare against each fall reporting strategy in isolation and in combination.

#### Sensitivity Analysis

The above analyses were repeated for feasibility, proportion of missing data, and accuracy separated by trial arm (Tai Chi or control group).

## RESULTS

Of 83 dyads randomized, 13 withdrew during the trial. All data available up to withdrawal were included in the analysis. Descriptive characteristics of PWD are shown in Table S1 (Supplemental Digital Content 1, http://links.lww.com/WAD/A256).

### Feasibility of Falls Data Collection

Table 1 presents the feasibility of each reporting method. The most feasible methods of falls data collection were weekly (84%) and 3-monthly telephone interviews (81%). Among 83 PWD, 37 experienced a fall, with a total of 116 falls after converging the different reporting methods. A further 28 falls with missing data was reported in telephone interviews (Table S2, Supplemental Digital Content 1, http://links.lww.com/WAD/A256).

**TABLE 1:** Feasibility of Each Data Collection Method (N=83)

	Expected	Obtained	%*	%†
Calendars‡	576	402	70	
Weekly telephone interviews§	2134	1803	84	
Weekly with PWD		1058		59
PWD making 75% or more of all weekly interviews				32 (n=27)
Weekly with carer		742		41
Carers making 75% or more of all weekly interviews				23 (n=19)
Weekly with unknown		3		<1
Monthly telephone interviews∥	576	426	74	
Monthly with PWD		242		57
PWD making 75% or more of all monthly interviews				40 (n=33)
Monthly with carer		182		43
Carers making 75% or more of all monthly interviews				29 (n=24)
Monthly with unknown		2		<1
3-monthly telephone interviews with carers¶	150	122	81	

*Proportion of the collected volume of data for each method to the data expected by each method.

†Proportion of the collected volume of data by person reporting falls (PWD, carers or unknown) by each method to the all data collected by each method.

‡Of 576 expected, 402 were returned (70%), of which 93% were valid. Thus, 65% of calendars were returned and valid. In total, 16% of participants returned all their expected calendars that were also valid.

§35% of participants completed all their expected weekly interviews.

∥Three dyads completed all their expected monthly interviews.

¶94% of participants completed all their expected 3-monthly interviews.

PWD indicates people with dementia.

### Accuracy of Fall Reporting

The numbers of falls reported by each method and their combinations are shown in Table 2. As a single method, calendars recorded the largest number of falls (62%) followed by weekly telephone interviews (59%). Each method in isolation missed at least 38% of the total falls reported. For combinations of methods, calendars and weekly telephone interviews had the highest accuracy (96%).

**TABLE 2:** Falls Captured by Each Data Collection Strategy and Combinations of Methods

	Total Falls (N=116)
	Count (%*)
Calendar	72 (62)
Weekly interviews total	69 (59)
With PWD	45 (39)
With carer	24 (21)
Monthly interviews total	26 (22)
With PWD	12 (10)
With carer	14 (12)
3-monthly interviews with carers	10 (9)
Calendar+weekly interviews	111 (96)
Calendar+monthly interviews	78 (67)
Calendar+3-monthly interviews	73 (63)
Weekly+monthly interviews	74 (64)
Weekly+3-monthly interviews	70 (60)
Monthly+3-monthly interviews	27 (23)

*Proportion of falls reported by each method to the total number of confirmed falls.

PWD indicates people with dementia.

### Sensitivity Analysis

The descriptive statistics indicated similar feasibility and accuracy of fall reporting between the intervention and control arms of the trial (Tables S3-5, Supplemental Digital Content 1, http://links.lww.com/WAD/A256). However, just under a quarter of falls reported by the control group had missing data (22/94) versus 12% of those in the Tai Chi group (6/50).

## DISCUSSION

To our knowledge, this is the first study to compare different methods of collecting falls data on community-dwelling PWD. The combination of daily calendars returned monthly and weekly telephone interviews produced the lowest level of missing data and highest accuracy of falls.

### Feasibility of Fall Recording Methods

Contrary to previous research that used more stringent criteria for assessing the validity of returned calendars,^4^ where only 60% of returned calendars were valid, we found that 93% of returned fall calendars were valid. A possible barrier to using calendars among PWD is forgetting to complete them and the inconvenience of returning them by post.^8^

### Accuracy

The combination of calendars and weekly telephone interviews recorded more falls than any other method. Each method in isolation had poorer accuracy. Moreover, monthly interviews with PWD produced more missing data than accurate data, especially in the control group. It has been reported that recall of falls was more accurate in an intervention group.^9^ Perhaps intervention groups are better able to recall falls due to a more structured weekly routine.

### Implications for Evaluations of Fall Prevention Interventions With PWD

The combined use of daily calendars returned monthly with weekly telephone interviews were a superior method because of the greater accuracy (but lower feasibility) of calendars along with greater feasibility (but lower accuracy) of weekly interviews. Future evaluations should use this approach and allow carers to provide data as well. These results raise concern about the existing evidence base on interventions to prevent falls among PWD as the majority of trials have relied on monthly reporting. While it is recommended to collect falls data from the general older population using daily calendars returned monthly, and to collect any further/missing data by interview monthly,^3^ such an approach with PWD is at risk of higher levels of missing data and inaccuracy that can be avoided in future.

### Study Limitations and Ideas for Future Research

The study period was for 6 months. Future research could assess longer-term feasibility and accuracy of fall reporting among PWD and assess how much assistance PWD receive in completing fall calendars.

## CONCLUSION

Evaluations of interventions to prevent falls among PWD should use a combination of calendars and weekly telephone interviews where possible to provide more complete and accurate data.

## Supplementary Material

SUPPLEMENTARY MATERIAL

Supplemental Digital Content is available for this article. Direct URL citations appear in the printed text and are provided in the HTML and PDF versions of this article on the journal's website, www.alzheimerjournal.com.
