# Supplementary material for: Feasibility and Accuracy of Different Methods for Collecting Data on Falls Among Older People With Dementia
Source: Alzheimer Dis Assoc Disord. 2019 Nov 26;34(4):362–5. doi: 10.1097/WAD.0000000000000364 (PMC7116406; doi:10.1097/WAD.0000000000000364)
Supplement: SUPPLEMENTARY MATERIAL [file wad-34-362-s001.docx]

**Feasibility and accuracy of different methods for collecting data on falls among older people with dementia**

**Supplemental Digital Content**

Page 2**:** Table S1: Baseline demographic characteristics of people with dementia and their informal carers.

Page 4**:** Table S2: Proportions of falls with and without missing data captured by telephone interviews.

Page 5**:** Table S3. Feasibility of fall reporting between the intervention and control groups of the trial.

Page 6**:** Table S4. Falls captured by each method of data collection between the intervention and control groups of the trial.

Page 7**:** Table S5. Falls with and without missing data in total and in comparison with the control group and intervention group.

Table S1. Baseline demographic characteristics of people with dementia and their informal carers.

| Characteristic (N = 83) | Statistic | Value |
| --- | --- | --- |
| *People with dementia* |  |  |
| Age (years) | Mean (SD) | 77.44 (7.95) |
| White ethnicity | n (%) | 81 (98%) |
| Male / female | n (%) | 50 (60%) / 33 (40%) |
| Education |  |  |
| None | n (%) | 3 (4%) |
| Primary school | n (%) | 2 (2%) |
| Secondary school | n (%) | 45 (54%) |
| Higher education, college, university | n (%) | 24 (29%) |
| Further education, professional qualification | n (%) | 9 (11%) |
| Relationship status |  |  |
| Single | n (%) | 2 (2%) |
| Married | n (%) | 68 (82%) |
| With partner | n (%) | 1 (1%) |
| Divorced | n (%) | 2 (2%) |
| Widowed | n (%) | 10 (12%) |
| Living situation |  |  |
| Living alone | n (%) | 7 (8%) |
| Living with family or friends | n (%) | 76 (92%) |
| Mini Addenbrooke’s Cognitive Examination score | Mean (SD) | 15.73 (4.63) |
| Time since dementia diagnosis (months) | Median (range) | 14.5 (107) |
| Dementia diagnosis |  |  |
| Alzheimer’s | n (%) | 56 (68%) |
| Vascular | n (%) | 6 (7%) |
| Mixed Alzheimer’s and Vascular | n (%) | 15 (18%) |
| Other | n (%) | 6 (7%) |
| *Informal carers* |  |  |
| Age (years) | Mean (SD) | 70.67 (10.09) |
| Male / female | n (%) | 17 (20%) / 66 (80%) |
| Living with the person with dementia | n (%) | 72 (87%) |
| Spouse / partner of the person with dementia | n (%) | 65 (78%) |

*Eligibility criteria for informal carers*: Informal carers were able to: commit to supporting the PWD with data collection throughout the trial and in the intervention components if allocated to the intervention group, physically able to do standing Tai Chi, and willing to attend weekly Tai Chi classes. Carers were excluded if they had severe sensory impairment or lacked mental capacity to provide informed consent.

Table S2: Proportions of falls with and without missing data captured by telephone interviews.

|  | Falls with dates | | Falls with missing date data that were excluded | |
| --- | --- | --- | --- | --- |
|  | N = 116 | | N = 28 | |
|  | Count¹ | Ratio² | Count¹ | %³ |
| Weekly interviews total | 69 | 0.04 | 3 | 11% |
| With PWD | 45 | 0.04 | 2 | 7% |
| With carer | 24 | 0.04 | 1 | 4% |
| Monthly interviews total | 26 | 0.61 | 16 | 57% |
| With PWD | 12 | 1.25 | 15 | 54% |
| With carer | 14 | 0.07 | 1 | 4% |
| 3-monthly interviews with carers | 10 | 0.90 | 9 | 32% |
| Weekly + Monthly interviews | 74 | 0.26 | 19 | 68% |
| Weekly + 3-monthly interviews | 70 | 0.17 | 12 | 43% |
| Monthly + 3-monthly interviews | 27 | 0.93 | 25 | 89% |
| PWD = people with dementia  ¹ Number of falls with data recorded by a given method  ² For each method, the ratio of falls with missing data: falls without missing data  ³ Percentage using each method amongst 28 with missing falls date data | | | | |

Table S3. Feasibility of fall reporting between the intervention and control groups of the trial.

|  | Control group = 41 | | | | Intervention group = 42 | | | |
| --- | --- | --- | --- | --- | --- | --- | --- | --- |
|  | Exp | Rec | %^1^ | %^2^ | Exp | Rec | %^1^ | %^2^ |
| Calendars | 280 | 214 | 76% |  | 296 | 188 | 63% |  |
| Weekly interviews | 1032 | 864 | 83% |  | 1102 | 939 | 85% |  |
| With PWD |  | 521 |  | 60% |  | 537 |  | 57% |
| With carer |  | 342 |  | 40% |  | 400 |  | 43% |
| Unknown |  | 1 |  | <1% |  | 2 |  | <1% |
| Monthly interviews | 280 | 205 | 73% |  | 296 | 221 | 75% |  |
| With PWD |  | 111 |  | 56% |  | 131 |  | 60% |
| With carer |  | 93 |  | 47% |  | 89 |  | 40% |
| Unknown |  | 1 |  | <1% |  | 1 |  | <1% |
| 3-monthly interviews with carers | 72 | 58 | 80% |  | 78 | 64 | 82% |  |
| PWD = people with dementia  Exp = Expected  Rec = Received  ¹ Proportion of the collected volume of data for each method to the data expected by each method.  ² Proportion of the collected volume of data by person reporting falls (PWD, carers or unknown) by each method to the all data collected by each method. | | | | | | | | |

Table S4. Falls captured by each method of data collection between the intervention and control groups of the trial.

| Period | Total | | Control group | | Intervention group | |
| --- | --- | --- | --- | --- | --- | --- |
| Number of falls | N = 116 | | N = 72; 62% | | N = 44; 38% | |
|  | Count | % | Count | % | Count | % |
| Calendar | 72 | 62% | 42 | 58% | 30 | 68% |
| Weekly interviews total | 69 | 59% | 40 | 55% | 29 | 66% |
| PWD | 45 | 39% | 29 | 40% | 16 | 36% |
| Carer | 24 | 21% | 11 | 15% | 13 | 29% |
| Monthly interviews total | 26 | 22% | 15 | 21% | 11 | 25% |
| PWD | 12 | 10% | 8 | 11% | 4 | 9% |
| Carer | 14 | 12% | 7 | 10% | 7 | 16% |
| 3-monthly interviews with carers | 10 | 9% | 4 | 5% | 6 | 14% |
| Calendar + weekly interviews | 111 | 96% | 67 | 93% | 44 | 100% |
| Calendar + monthly interviews | 78 | 67% | 44 | 64% | 34 | 77% |
| Calendar + 3-monthly interviews | 73 | 63% | 41 | 60% | 32 | 73% |
| Weekly + monthly interviews | 74 | 64% | 45 | 60% | 29 | 66% |
| Weekly + 3-monthly interviews | 70 | 60% | 42 | 55% | 28 | 63% |
| Monthly + 3-monthly interviews | 27 | 23% | 17 | 23% | 10 | 23% |
| PWD = people with dementia  ¹ Proportion of falls reported by each method to the total number of confirmed falls | | | | | | |

Table S5. Falls with and without missing data in total and in comparison with the control group and intervention group.

|  | Falls without missing data | | Falls with missing data | | | | | |
| --- | --- | --- | --- | --- | --- | --- | --- | --- |
|  |  | |  | | Control group | | Intervention group | |
|  | N = 116 | | N = 28 | | N = 22 | | N = 6 | |
|  | Count | %^1^ | Count | %^2^ | Count | %^3^ | Count | %^3^ |
| Weekly interviews | 69 | 6% | 3 | 11% | 3 | 100% | 0 | 0% |
| With PWD | 45 | 7% | 2 | 7% | 2 | 100% | 0 | 0% |
| With Carer | 24 | 4% | 1 | 4% | 1 | 100% | 0 | 0% |
| Monthly interviews | 26 | 65% | 16 | 57% | 13 | 81% | 3 | 20% |
| With PWD | 12 | 125% | 15 | 54% | 13 | 87% | 2 | 13% |
| With Carer | 14 | 14% | 1 | 4% | 0 | 0% | 1 | 100% |
| 3-monthly interviews with carers | 10 | 90% | 9 | 32% | 6 | 67% | 3 | 33% |
| PWD = people with dementia  ^¹^Percentage of falls with missing data in each method relative to the number of confirmed falls reported by each method  ² Percentage of falls with missing data in each method relative to the total number of falls with missing data  ^3^ Percentage of falls with missing data in each method in each trial arm relative to the total number of falls with missing data | | | | | | | | |
